# Body satisfaction and body weight: gender differences and sociodemographic determinants

**DOI:** 10.1186/1471-2458-9-313

**Published:** 2009-08-27

**Authors:** S Bryn Austin, Jess Haines, Paul J Veugelers

**Affiliations:** 1 Division of Adolescent and Young Adult Medicine, Children's Hospital, Boston, USA; 2 Department of Society, Human Development and Health, Harvard School of Public Health, Boston, USA; 3 Department of Ambulatory Care and Prevention, Harvard Medical School, Harvard Pilgrim Health Care, Boston, USA; 4 School of Public Health, University of Alberta, Edmonton, Alberta, Canada

## Abstract

**Background:**

Given the documented links between body satisfaction, weight-related behaviors, and weight change in adolescents, we sought to examine the prevalence of poor body satisfaction in prepubescent girls and boys and its associations with body weight, socioeconomic factors, and rural residence.

**Methods:**

We obtained data from 4254 girls and boys participating in a population-based survey of grade five students in the province of Nova Scotia, Canada. We examined gender specific associations between the prevalence of poor body satisfaction and body mass index (BMI) with generalized additive models and applied multilevel logistic regression methods to estimate associations of body satisfaction with BMI, rural residence, parental education and income, and neighborhood household income.

**Results:**

We observed a linear increase in poor body satisfaction with increasing BMI in girls. Among boys, however, we found a U-shape association where boys with low BMI and those with high BMI reported higher levels of poor body satisfaction. We also found that poor body satisfaction was more prevalent among girls whose parents had lower educational attainment and among those who reside in rural areas.

**Conclusion:**

Insight into the unique relationships between body satisfaction and BMI experienced by prepubescent children, males, and populations diverse in parental education and geographic location may help to inform public health initiatives designed to improve weight-related behaviors and reduce overweight in children.

## Background

A rapid rise in childhood overweight over the past two decades, now estimated to be as high as 26% in children 6 to 11 years in Canada [1] and 33% in children of the same ages in the United States [2], has prompted redoubled efforts to identify drivers of the increases and key leverage points at which to target preventive interventions. Emerging evidence suggests that body satisfaction may be such a leverage point.

The relationship between poor body satisfaction and increased risk of onset of disordered weight control behaviors and symptoms, including vomiting, fasting, and use of laxatives and diet pills for weight control, has been well-established in prospective studies with adolescent females and males [3-5]. Beyond its links with eating disorder symptoms, body satisfaction has captured the attention of researchers and interventionists because of its potential role in efforts to prevent childhood overweight and promote healthful nutrition and physical activity. Recent findings from the Minnesota-based Project EAT study have provided important insights in this regard. In Project EAT, a community-based, observational cohort of over 2500 girls and boys first enrolled in the study when in junior and senior high school, Neumark-Sztainer *et al*. found greater body satisfaction at baseline was associated with more healthful dietary and physical activity behavior at follow-up five years later, when participants were in late adolescence and young adulthood [4]. In the same cohort, Haines *et al*. found that in both girls and boys, those with greater body satisfaction at baseline were less likely to be overweight at follow-up five years later [6]. Furthermore, analyzing Project EAT data from the subset of 376 girls who were already overweight at baseline, van den Berg *et al*. found that higher body satisfaction at baseline predicted less weight gain over five years of follow-up [7].

Importantly, body satisfaction appears to be mutable, as school-based interventions have achieved modest improvements in body satisfaction in both girls and boys [8-10]. Targeting interventions to promote body satisfaction in prepubescent children may have several advantages for overweight prevention unique to this developmental period. One, studies have found body satisfaction declines with the onset of adolescence in both males [11,12] and females [11-13]. Two, for the majority of children with healthful body weights, interventions that help them adopt and maintain healthful weight-related behaviors will support primary prevention. In fact, a large proportion of overweight adults may not have been overweight as children; therefore, population-based primary prevention to instill healthy behaviors in non-overweight children may have a lasting impact on prevention of overweight in adulthood [14], And three, timing interventions to precede the completion of normal growth (height velocity peaks at approximately age 12 years in girls and 14 years in boys [15]) may potentiate attenuation of BMI trajectory slopes without necessitating weight loss per se (i.e., weight loss measured in kilograms or pounds). In this respect, one longitudinal, observational study of almost 6,000 children in kindergarten through 8^th ^grade in the Boston area found prepubescent children were more likely than older youth to experience remission of overweight over a one-year period [16].

Much research has been conducted on the relationship between body satisfaction and BMI in adolescents, adults, and female children, but less is known about the relationship in male children [17,18]. Body satisfaction has consistently been found to be higher in males than in females at all ages [17], and recent evidence suggests that gender may modify the relationship between BMI and body satisfaction [12,19]. For instance, in one Australian study with over 900 children and adolescents, Kostanski *et al*. found a linear increase in body dissatisfaction with increasing BMI in females, but in males, both those with very low BMI and those with high BMI reported more body dissatisfaction than did boys in the healthy BMI range [12]. In addition, low socioeconomic position (SES) [20] and rural residence [21-23] have been linked with higher BMI in children, but how these factors may pattern body satisfaction in children is unclear. Social norms regarding ideal weight and body size may differ by socioeconomic position [24] and geographic residence. To increase our understanding of body satisfaction and its links with BMI in childhood, we studied the prevalence of poor body satisfaction in prepubescent girls and boys. Furthermore, to provide direction for research and preventive policy, we studied the associations of poor body satisfaction with body weight, socioeconomic factors, and rural residence.

## Methods

### Study design

We obtained data from the 2003 Children's Lifestyle and School-performance Study (CLASS), a survey of grade five students, who are primarily 10 or 11 years old, in the province of Nova Scotia, Canada [25,26]. In Nova Scotia, over 95% of residents are of European decent and 98.4% of students attend public schools. Of all 291 public schools in Nova Scotia, 282 participated with an average student participation rate of 51% per school. Study representatives visited schools to administer a questionnaire and to measure the height and weight of students for whom parental consent was obtained [25]. Standing height was measured to the nearest 0.1 cm after students had removed their shoes and body weight to the nearest 0.1 kg on calibrated digital scales. Height and weight were used to calculate the BMI (weight in kilograms divided by height in meters squared). Overweight and obesity were classified using the International Obesity Task Force sex- and age-specific standards for children [27]. This study was approved by the Human Research Ethics Board at Dalhousie University in Halifax, Nova Scotia, Canada.

In the present study we used the question "I like the way I look" as a proxy for body satisfaction. Response choices included "never or almost never," "sometimes" to "often or almost always." We coded students responding with "never or almost never" as having a poor body satisfaction and students with other responses not having poor body satisfaction. Parents completed a survey that included questions on parental education and household income. We estimated neighborhood income by averaging, per school, the postal-code level of income (available through the 2001 Canada census) of residential addresses of children attending the school.

### Statistical Analysis

We examined the association between the prevalence of poor body satisfaction and BMI with generalized additive models (GAM) [28]. GAM relaxes the usual assumption of linearity in regression analyses to enable researchers to uncover other, non-linear, patterns. GAM generates flexible smoothed curves of the association with 95% confidence intervals that further facilitates the judgment of linearity. We also tested linearity using logistic regression models for the probability of poor body satisfaction with BMI and quadratic and 3^rd ^order polynomial functions of BMI as independent variables. Statistically significant presence of quadratic or 3^rd ^order polynomial BMI functions would indicate non-linearity of BMI in its association with body satisfaction. As we work with hierarchical data whereby observations of students and their parents are nested within that of schools, we applied multilevel logistic regression methods. We considered BMI and quadratic and 3^rd ^order polynomial functions of BMI as well as the potential confounders, parental educational attainment and household income, as first level variables and considered neighborhood level confounders, rural or urban residency and neighborhood level income, as a second level variable [29].

A total of 5200 students were surveyed. As one of the seven school boards did not allow measurements of height and weight, BMI is available for 4298 students. Of these students, 44 (1%) did not complete the body satisfaction question leaving a total 4254 students with complete information on both BMI and body satisfaction, 2159 of which were girls and 2095 boys. For these students, 311 (7.3%) had missing information on parental education and 963 (22.6%) on household income, which was an elective question. These missing values were considered as a missing category in the statistical analyses. Prevalence estimates were weighted to reflect prevalence estimates of that of the provincial population of 10- and 11-year-old children [25]. All analyses were conducted using S-Plus version 7 (Insightful Corp., Seattle, WA, USA) and HLM version 6 (Scientific Software International, Lincolnwood, IL, USA).

## Results

Among grade five students in Nova Scotia, 7.3% of girls and 7.8% of boys reported poor body satisfaction (Table 1). For normal weight, overweight and obese girls the prevalence of poor body satisfaction was 5.7%, 10.4% and 13.1% respectively. For boys this was 7.6%, 8.4%, and 8.1% respectively (Table 1).

**Table 1:** Characteristics of 10- and 11-year-old girls and boys in Nova Scotia, Canada

	Girls (n = 2159)	Boys (n = 2095)
**Poor body satisfaction (%)**	7.3	7.8

		

**Overweight (%)**	33.0	33.1

**Obesity (%)**	9.0	10.9

		

**Poor body satisfaction:**		

Among normal weight students (%)	5.7	7.6

Among overweight students (%)	10.4	8.4

Among obese students (%)	13.1	8.1

		

**Parental education**:		

Secondary or less (%)	32.3	27.5

Community college (%)	37.0	38.3

University (%)	21.8	24.7

Graduate university (%)	8.9	9.5

		

**Annual household income**:		

< $20,000 (%)	10.7	10.8

$20,000‐$40,000 (%)	23.7	21.3

$40,000‐$60,000 (%)	25.2	27.4

> $60,000 (%)	40.4	40.5

		

**Residency**:		

Rural (%)	37.9	38.7

Urban (%)	62.1	61.3

**Figure 1:**
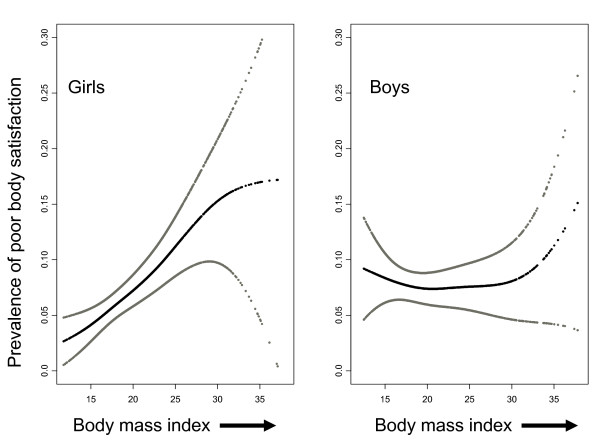
**Prevalence of poor body satisfaction by body mass index among 10- and 11-year-old girls and boys in Nova Scotia, Canada**. Black lines: estimated prevalence; grey lines: 95% confidence intervals

Figure 1 visualizes the distinct associations between the prevalence of poor body satisfaction and BMI that exist for girls and boys. The linearity of this association for girls was confirmed by the observation that quadratic and polynomial functions of BMI did not contribute in a statistically significant way in our analyses. One unit of increase in BMI for girls was associated with an 8.1% higher prevalence of poor body satisfaction (Table 2). The U-shape associations of the prevalence of poor body satisfaction with BMI for boys (Figure 1) was confirmed by the observation that the squared value of BMI contributed in a statistically significant way to the model presented in Table 2. Table 2 also shows that girls from parents with low educational attainment and residing in rural areas are more likely to report poor body satisfaction.

**Table 2:** Associates of poor body satisfaction among 10- and 11-year-old girls and boys in Nova Scotia, Canada^a^

	Girls **Odds Ratio 95%CI**^**b**^	Boys **Odds Ratio 95% CI**^**b**^
**Body mass index**	1.081 (1.043, 1.119)	0.820 (0.674,0.998)

**Body mass index squared**		1.004 (1.000, 1.008)

		

**Parental education**:		

Secondary or less	1	1

Community college	0.566 (0.405, 0.792)	0.839 (0.564, 1.249)

University	0.581 (0.342, 0.988)	0.687 (0.435, 1.085)

Graduate university	0.640 (0.286, 1.431)	0.484 (0.228, 1.026)

		

**Annual household income**:		

< $20,000	1	1

$20,000‐$40,000	0.977 (0.539, 1.769)	1.231 (0.645, 2.246)

$40,000‐$60,000	0.886 (0.506, 1.551)	0.977 (0.491, 1.946)

> $60,000	0.724 (0.388, 1.351)	0.646 (0.328, 1.271)

		

**Residency**:		

Rural	1	1

Urban	0.686 (0.472, 0.999)	1.017 (0.701, 1.476)

		

**Neighborhood income**:		

Lowest one third	1	1

Middle one third	0.852 (0.556, 1.328)	1.049 (0.665, 1.656)

Highest one third	1.264 (0.824, 1.937)	1.193 (0.757, 1.881)

^a ^All estimates were obtained through gender-stratified multilevel multivariate logistic regression models; ^b ^CI = confidence interval.

## Discussion

Body satisfaction is emerging as a potentially valuable leverage point for public health efforts to address childhood overweight for two primary reasons: High body satisfaction has been prospectively linked with healthful weight-related behaviors and reduced weight gain [4-6], and it has been shown to be modifiable in school-based interventions [8-10]. While substantial research has focused on body satisfaction in some subgroups, namely female adolescents and adults, advances in population-based overweight prevention efforts in children will require greater insight into the unique experiences of prepubescent children, males, and populations diverse in SES and geographic location. Our findings contribute to the literature by examining patterns in body satisfaction and BMI within subgroups of children who have received little research attention on these issues, especially males and children in rural areas and those from families of low parental education. In our school-based study of over 4000 Canadian preadolescents, we found a linear increase in poor body satisfaction with increasing BMI in girls. Among boys, however, we found a U-shape association where boys with low BMI and those with high BMI reported higher levels of poor body satisfaction. We also found that poor body satisfaction was more prevalent among girls whose parents had lower educational attainment and among those who reside in rural areas.

Our results are in agreement with one previous study that found a positive linear association between body dissatisfaction and BMI among girls and a U-shape association among boys in a sample of over 900 Australian children and adolescents ranging in age from 7 to 18 years [12]. Poor body satisfaction among males with a low BMI may reflect the cultural ideal for males to attain both muscularity and leanness [30]; whereas, among females, thinness remains the culturally defined ideal body shape [31]. Our finding that girls from parents with low educational attainment were more likely to report poor body satisfaction is similar to that of Robinson and colleagues, who found that parental educational attainment was negatively associated with body dissatisfaction among white third grade girls in California [24]. Interestingly, Robinson *et al*. did not find an association between body satisfaction and parental education among African American girls or among boys, suggesting that the associations may differ by race/ethnicity and gender. In the 24-country Health Behaviour of School-Aged Children study, Al Sabbah et al. found in Canadian youth ages 11, 13, and 15 years old that difficulty communicating with both their father and mother was associated with increased risk of body weight dissatisfaction in girls [32]. It is possible that problems with family communication may be one factor underlying the observed association in our Nova Scotia sample between low parental educational attainment and poor body satisfaction in girls.

Our examination of body satisfaction by urban/rural geographic residence among Canadian youth is novel. Our finding that girls who reside in rural areas, controlling for BMI, are more likely than urban girls to report poor body satisfaction may suggest that body or appearance-related pressures are higher within rural areas or perhaps that girls in urban areas benefit from existing community, school, or other programs that may protect against decrements in body satisfaction. Additional research is needed to elucidate how weight-related norms and pressures differ by geographic residence and how these norms may differ by gender. Residual confounding by BMI may also be an explanation for this finding, as rural Canadian youth are more likely to be overweight or obese as compared to urban youth [21,23].

Strengths of our study include the examination of body satisfaction among a large, population-based sample of preadolescents from schools that are diverse with regards to geographic location of residence, neighborhood median household income, and parental education and income. Our examination of contextual factors related to body satisfaction in children using multilevel data and analytic methods is novel. Other strengths include direct measurements of participants' height and weight and adjustment for nonresponse bias and near full participation of elementary schools in the province (282/291 elementary schools in Nova Scotia). Some limitations should be considered however. The study population is predominantly white and restricted to one region of the nation, which limits the generalizability of our findings. An additional limitation of this study is that the data were cross-sectional. Further, we used a single-item indicator of body satisfaction, which may have reduced reliability and validity of measurement relative to multi-item instruments [33,34]. We recommend the present findings be confirmed in a longitudinal study using a multi-item instrument.

## Conclusion

In sum, we found that the association between body satisfaction and BMI differs by gender among prepubescent children. We also found that, among girls, lower parental education and living rurally is associated with poorer body satisfaction. Given the links between body satisfaction, weight-related behaviors, and weight gain in youth, public health initiatives for overweight prevention with children may be strengthened through better understanding of factors underlying gender differences in body satisfaction and the mechanisms by which living in families with low parental education and in rural communities contribute to poorer body satisfaction among preadolescent girls. In addition, with the substantial prevalence of poor body satisfaction, public health initiatives designed to improve body satisfaction along with promotion of healthy eating and active living in children as young as 10 and 11 years are appropriate and warranted.

## Competing interests

The authors declare that they have no competing interests.

## Authors' contributions

SBA contributed to conception of the study question, analysis approach, data interpretation, and manuscript drafting and critical revision. JH contributed to conception of the study question, analysis approach, data interpretation, and manuscript drafting and critical revision. PJV designed the study, collected the data, performed statistical analyses, and contributed to manuscript drafting and critical revision. All authors read and approved the final manuscript.

## Pre-publication history

The pre-publication history for this paper can be accessed here:

http://www.biomedcentral.com/1471-2458/9/313/prepub
